# Metabolism makes and mends the heart

**DOI:** 10.7554/eLife.54665

**Published:** 2020-02-04

**Authors:** Megan L Martik

**Affiliations:** Division of Biology and Biological EngineeringCalifornia Institute of TechnologyPasadenaUnited States

**Keywords:** metabolism, heart development, regeneration, cardiomyocytes, Mouse, Zebrafish

## Abstract

Experiments in zebrafish have shed new light on the relationship between development and regeneration in the heart.

**Related research articles** Fukuda R, Aharonov A, Ong YT, Stone OA, El-Brolosy M, Maischein H-M, Potente M, Tzahor E, Stainier DYR. 2019. Metabolic modulation regulates cardiac wall morphogenesis in zebrafish. *eLife* **8**:e50161. Honkoop H, de Bakker DEM, Aharonov A, Kruse F, Shakked A, Nguyen PD, de Heus C, Garric L, Muraro MJ, Shoffner A, Tessadori F, Peterson JC, Noort W, Bertozzi A, Weidinger G, Posthuma G, Grün D, van der Laarse WJ, Klumperman J, Jaspers RT, Poss KD, van Oudenaarden A, Tzahor E, Bakkers J. 2019. Single-cell analysis uncovers that metabolic reprogramming by ErbB2 signaling is essential for cardiomyocyte proliferation in the regenerating heart. *eLife* **8**:e50163.

The human heart is a remarkable organ, but the zebrafish heart is even more remarkable because it can repair itself if it is damaged. This repair process, which is known as regeneration, has much in common with the complex developmental processes by which the heart is made in zebrafish embryos. In the human heart, injury such as a heart attack leads to scarring and ultimately heart failure, so a better understanding of the links between heart development and regeneration in zebrafish could help with efforts to improve the efficiency of regeneration in humans. Two papers in eLife may help with these efforts by showing that metabolism has a role in both processes.

During development, cardiomyocytes – the cells of that make up the heart muscle called the myocardium – undergo complex movements that allow the inner walls of the heart to form (Figure 1A; Moorman and Christoffels, 2003; Staudt and Stainier, 2012). These inner walls, also known as trabeculae, are muscular ridges that help the heart to contract and also help to oxygenate the developing cardiac wall (Sedmera et al., 2000). In one of the eLife papers, Ryuichi Fukuda, Didier Stainier (both at the Max Planck Institute for Heart and Lung Research), and colleagues report the results of experiments that used 3D single-cell imaging, cell transplantation, and genetic techniques to study the development of trabeculae (Fukuda et al., 2019). They show that cardiomyocytes undergo extensive changes in shape as they separate, or delaminate, from the myocardium to form trabeculae.

**Figure 1.**
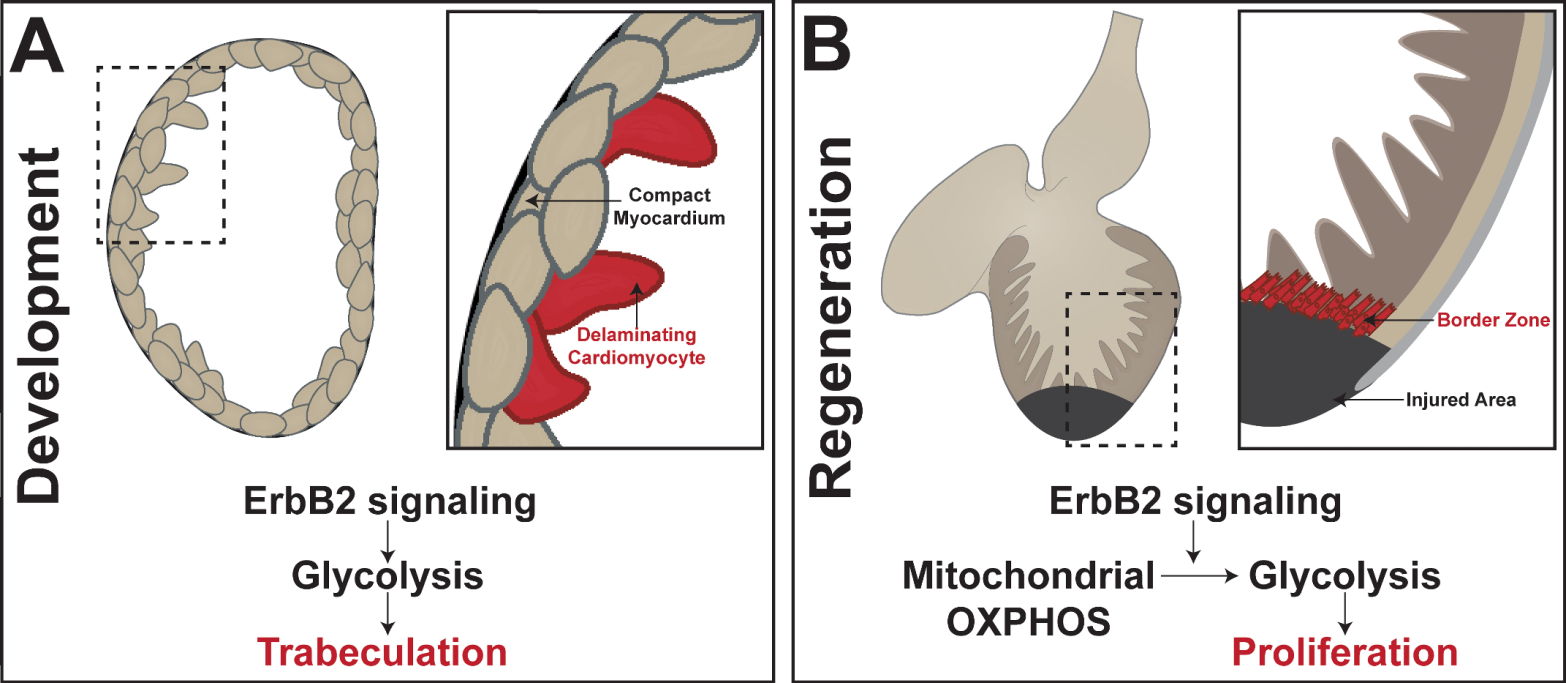
Metabolic control of heart development and adult heart repair in the zebrafish. (**A**) Trabeculae form in the heart when compact cardiomyocytes delaminate from the myocardium. Fukuda et al. found that Nrg1/ErbB2 signaling activates glycolysis to initiate the complex cell movements involved in delamination. (**B**) In the regenerating adult zebrafish heart, cardiomyocytes in the 'border zone' at the edge of the injured area de-differentiate and proliferate. Honkoop et al. uncovered a role for Nrg1/ErbB2 signaling in the control of metabolic reprogramming of the border zone cardiomyocytes from oxidative phosphorylation (OXPHOS) to glycolysis. Further, they found that glycolysis is required for proliferation after injury.

Previous work had shown that the Nrg1/ErbB2 signaling pathway was involved in the formation of trabeculae (Lee et al., 1995; Liu et al., 2010), and Fukuda et al. now show that this pathway also activates glycolysis to initiate the delamination process (Figure 1A). In particular, they show that cells with suppressed glycolytic activity fail to undergo the complex cell movements needed for the proper formation of trabeculae. While much is known about metabolic processes in the adult heart, Fukuda et al. reveal a previously understudied role for glycolysis during heart development.

In the adult heart, cardiomyocytes rely on fatty acid metabolism and mitochondrial oxidative phosphorylation as their primary sources of metabolic activity. In mice, cardiomyocytes undergo a metabolic shift from glycolysis to oxidative phosphorylation in the first post-natal week, and this correlates with these cells losing their ability to proliferate (Lopaschuk et al., 1992; Menendez-Montes et al., 2016). Since it is known that the mouse heart loses the ability to regenerate within a week of birth, it is intriguing to think that the metabolic shift would have a role in suppressing the ability to repair (Porrello et al., 2011).

Zebrafish, on the other hand, retain their remarkable capacity to regenerate into adulthood. In the injured zebrafish heart, cardiomyocytes near the injured area, or 'border zone', dedifferentiate and then proliferate to contribute to regeneration (Figure 1B; Poss et al., 2002; Kikuchi et al., 2010; Jopling et al., 2010). However, the heterogeneity of cell types found in the heart during repair has complicated efforts to fully understand the regenerative ability of zebrafish.

In the second paper, Jeroen Bakkers (Hubrecht Institute and University Medical Center Utrecht) and colleagues – including Hessel Honkoop and Dennis de Bakker as joint first authors – report how they used a newly generated transgenic reporter line that labels border zone cardiomyocytes and a technique called fluorescence activated cell sorting to obtain pure populations of both proliferating border zone cells and non-proliferating 'remote cells' (Honkoop et al., 2019). Individual cells from both populations then underwent single-cell RNA sequencing, as did embryonic cardiomyocytes. Intriguingly, this revealed that the proliferating border zone cells resembled the embryonic cells more than they resembled the non-proliferating remote cells.

Honkoop et al. further showed that border zone cardiomyocytes differ from remote cardiomyocytes in that oxidative phosphorylation is reduced while glycolysis and lactate fermentation is increased. This suggests that the border zone cells undergo metabolic reprogramming to have a more embryonic-like metabolic state. Honkoop et al. also investigated the Nrg1/ErbB2 signaling pathway as previous research had shown that this pathway was necessary for cardiomyocyte proliferation after injury (D'Uva et al., 2015; Gemberling et al., 2015). They found that the pathway was induced in the border zone cells to regulate the shift to a glycolytic mechanism in adult cardiomyocytes. Moreover, when glycolysis was blocked, regeneration failed due to a down-regulation in proliferation. Honkoop et al. also showed that glycolysis genes are enriched in mouse cardiac tissue in which ErbB2 was over-expressed, and increased glycolytic activity improved repair in these mice after injury.

Overall, the results in these two papers contribute to our understanding of how regeneration reactivates developmental programs by activating a transcriptional profile similar to that found in embryonic populations and by using metabolic reprogramming to return cells to an embryonic-like state. Given that these mechanisms are conserved from fish to mammals, the data represent a promising therapeutic route for inducing human heart regeneration. However, we do not fully understand why the injured heart shifts to glycolysis in order to proliferate. Is it because proliferation is a high-energy process? Or is glycolysis necessary for other critical processes required for heart regeneration, such as complex changes of cell shape? Understanding the nuances of each process will be important as researchers look for ways to help the human heart regenerate after injury.
